# Response mechanism of growth and gypenosides content for *Gynostemma longipes* cultivated at two altitude habitats to fine root morphological characteristics

**DOI:** 10.3389/fpls.2023.1143745

**Published:** 2023-05-30

**Authors:** Doudou Li, Gang Li, Benye Xi, Jiaxia Gan, Dingmei Wen, Feng Cao, Fengmei Suo, Jincan Li, Baiping Ma, Baolin Guo

**Affiliations:** ^1^ Key Laboratory of Bioactive Substances and Resources Utilization of Chinese Herbal Medicines, Ministry of Education, Institute of Medicinal Plant Development, Chinese Academy of Medical Sciences and Peking Union Medical College, Beijing, China; ^2^ Beijing Institute of Radiation Medicine, Beijing, China; ^3^ Ministry of Education Key Laboratory of Silviculture and Conservation, Beijing Forestry University, Beijing, China; ^4^ Ankang Zhengda Pharmaceutical Co., Ltd., Ankang, China

**Keywords:** fine root, biomass, gypenosides, *Gynostemma longipes*, altitude habitat, soil factors

## Abstract

**Introduction:**

Fine roots are the critical functional organs of plants to absorb water and nutrients from the soil environment, while the relation between fine root morphological characteristics and yield & quality has received less attention for medicinal plants.

**Methods:**

Therefore, we investigated the relationship between fine root morphological characteristics and biomass & gypenosides content. We explored the primary environmental drivers of fine root indicators for *Gynostemma longipes* from three provenances cultivated at two altitude habitats.

**Results:**

At the end of the growing season, compared with the low-altitude habitat, the underground biomass of *G. longipes* in the high-altitude habitat increased significantly by 200%~290% for all three provenances. The response of gypenosides content to different altitude habitats varied with provenance and plant organs. The biomass of *G. longipes* strongly depended on the fine root characteristic indicators (*P* < 0.001), fine root length density, and fine root surface area. Our results also showed that the harvest yield of *G. longipes* could be effectively increased by promoting the growth of fine roots per unit leaf weight (*P* < 0.001, R^2^ = 0.63). Both fine root length density and fine root surface area had strong positive correlations with soil nutrient factors (R^2^ > 0.55) and a strong negative correlation with soil pH (R^2^ > 0.48). In a word, the growth of *G. longipes* is strongly controlled by the fine root morphological characteristics through the response of fine roots to soil nutrient factors and pH.

**Discussion:**

Our findings will help to deepen the understanding of the root ecophysiological basis driven by soil factors for the growth and secondary metabolites formation of *G. longipes* and other medicinal plants under changing habitat conditions. In future research, we should investigate how environmental factors drive plant morphological characteristics (e.g., fine roots) to affect the growth & quality of medicinal plants over a longer time scale.

## 1 Introduction

*Gynostemma* plants are well-known medicinal plants in oriental countries for a long history (Shaito et al., 2020; Su et al., 2021). Recently, *Gynostemma* plants have been widely used in the clinic due to their effects of lowering blood lipid, reducing blood glucose, and improving immunity, which has attracted significant attention (Gao et al., 2016; Huang et al., 2020; Su et al., 2021). Specifically, the dammarane-type saponins (gypenosides), the major biologically active constituents of *Gynostemma* plants and structurally correlated to ginsenosides (Nguyen et al., 2021; Ahmed et al., 2023). The wide application of *Gynostemma* plants in biopharmaceutical and healthy tea has dramatically increased the demand for its original materials. However, the research on precision cultivation of *Gynostemma* plants was relatively scarce. Therefore, the research on *Gynostemma* plants cultivation is essential for improving the yield and quality of *Gynostemma* plants and protecting wild resources. In addition, *G. Pentaphyllum* and *G. longipes* are the two most widely used species of *Gynostemma* plants, and the latter is more important than the former in medicinal efficacy (Wang et al., 2021). Therefore, *G. longipes* was selected as our research object.

It is well known that the growth and quality of medicinal plants are significantly affected by environmental factors. Iqbal et al. (2020); Iqbal et al. (2023) reported that phosphorus application in soil improved the cotton yield by enhancing reproductive organ biomass and nutrient accumulation in two cotton cultivars with different phosphorus sensitivity. Zahoor et al. (2017) investigated that potassium (K) application in the soil environment enhanced the cotton plant’s potential to maintain functionality under drought and facilitates recovery after rewatering. However, up to now, there has been no research report on the response of the growth and quality of *G. longipes* to environmental factors. There were only two studies on the response of the growth and quality of *G. pentaphyllum* to environmental factors. Zhang et al. (2005) showed that the increase of *G. pentaphyllum* production depended more on nitrogen and phosphorus in soil, while potassium was conducive to the accumulation of saponins in leaves and roots. Chang et al. (2016) indicated that the predicted increase in atmospheric temperature and CO_2_ could improve the biomass of *G. pentaphyllum*, but they would reduce its gypenosides content. These studies did not involve whether changes in altitude habitat will affect the growth and saponin content of *G. pentaphyllum*. Because the changes of environmental factors caused by altitude habitat are complex, such as the changes of soil physical and chemical properties (Defossez et al., 2018). Therefore, our first hypothesis is that changes in altitude habitat will significantly affect the growth and saponin content of *G. longipes*.

Fine roots are the hinge connecting plants and the soil environment and the critical organ for plants to absorb water and nutrient resources from soil. As an essential component of plant functional characteristics, fine root characteristics are crucial in explaining plant growth and development and metabolite accumulation. However, compared with the aboveground parts like leaves, branches, and stems, roots, as ‘the hidden half’ of plants, are more challenging to observe and measure and are less understood (Freschet et al., 2021). Some studies on crops or trees have shown that plant’s fine root characteristic parameters were closely related to plant growth and quality (Cui et al., 2019; Gao et al., 2019; Luo and Zhou, 2019; Yang and Kim, 2019). However, there is no research on the relationship between fine root morphological characteristics and plant growth and quality in medicinal plants. Therefore, our second hypothesis is that there is a quantitative relationship between the growth and quality formation of medicinal *G. longipes* and some parameters of fine root characteristics in different altitude habitats.

Many scholars have shown that fine root characteristics affect plant growth and quality formation because of environmental effects (Zhang et al., 2021; Lu et al., 2022). Soil is the crucial place for fine roots to absorb water and nutrients for plants to accumulate primary and secondary metabolites so that soil-related parameters may be one of the reasons for the differences in fine root characteristics of *G. longipes* in different habitats. Therefore, our third hypothesis is that there is a significant correlation between soil-related parameters and fine root characteristics of *G. longipes*.

Based on the above review, it is not yet clear whether the growth and saponins content of medicinal *Gynostemma* will be affected by changes in altitude habitat and how eco-physiological indicators (mainly fine root morphological characteristics) link plant growth & saponin content and habitat factors (soil physical and chemical properties). Therefore, the objectives of this study are (1) To clarify the growth and gypenosides characteristics of three provenances *G. longipes* at two altitude habitats; (2) To construct the relationship between growth & gypenosides and fine root morphological characteristics of *G. longipes*, and (3) To find out the main soil factors affecting the fine root morphology of *G. longipes*. To achieve these objectives, we measured the biomass, gypenosides, and fine root morphological characteristics of different organs for *G. longipes* from three provenances living in two altitude habitats. In addition, the soil physical and chemical properties of the two habitats were also determined. We hope our results help clarify the environmental mechanism of *G. longipes* growth and quality formation and help to enrich the theory of *G. longipes* genuine formation.

## 2 Materials and methods

### 2.1 Site description and experimental design

The study was conducted at Pingli County (31°37′ ~ 32°39′N and 109°~ 109° 33′E), Shaanxi Province, which is one of the natural distribution centers of *Gynostemma longipes* in China. The climate in this region is a subtropical humid monsoon climate, with an average air temperature of 14.5~15.7 °C and an annual frost-free period is 251 ~ 255 days. Our experimental sites were located in Dagui town with an altitude of 510 m, and Guangfo town with an altitude of 1150 m in Pingli County.

The experimental plantations were established in early April 2021 in two altitude areas, which were 510 m (H_1_) in Dagui town and 1150 m (H_2_) in Guangfo town, respectively. Three *G. longipes* provenances, which were Badao provenance from Shaanxi Province, Pingwu provenance from Sichuan Province, and Kangxian provenance from Gansu Province, were planted in H_1_ and H_2_ plantations. The plants were planted on the compartment about 1 meter wide by roots planting. The plant spacing was 30 cm, and the row spacing was 40 cm. They were planted in two experimental sites in early April with the same planting mode and followed the same field management measures during the growing seasons.

### 2.2 Plant growth and gypenosides assessment

At the end of the growing season in 2021, late October, plant samplings were obtained. As *G. longipes* is a climbing vine, all plants spread together in the early growth stage, so it was impossible to sample a single plant. So, we obtained the plant samples of different organs by delimiting a small sample square with a length and width of 30 cm and a depth of 20 cm (Freschet et al., 2021).

We randomly selected five small sample squares for each provenance at each altitude. So, for two altitudes and three provenances, a total of 30 small sample squares were obtained. We cut off the stems and leaves in each sample square with scissors, took them back to the laboratory for drying in the oven at 60°C for 48 h, and weighed them to estimate aboveground biomass (AB). The interconnected underground root system in each small sample square was returned to the laboratory and cleaned using flowing water. It was divided into the coarse root (diameter > 2mm) and fine root (diameter ≤ 2 mm) by vernier caliper. The coarse roots were directly dried, and the fine roots were dried after the scanning analysis (details in section 2.3) weighted to estimate underground biomass (UB). The total plant biomass (TPB) was the sum of AB and UB. AB, UB and TPB were used to evaluate the growth of *G. longipes* of three provenances cultivated at two altitude habitats.

For the above-dried leaf and root (fine root and coarse) samples, we crushed them to measure the gypenosides content. The gypenosides content in *G. longipes* was detected by ultrahigh performance liquid chromatography-charged aerosol detector (UHPLC-CAD). Precisely, accurately weigh 0.2 g of powder of *G. longipes* (passing 40 mesh sieve) and put it into a 100 ml conical flask with a stopper. Add 30 mL of 70% ethanol solution with a liquid material ratio of 1:150. The bottle stopper was tightly capped, shaken well, and ultrasonicated for 30 min. After cooling to room temperature, make up the weight loss. Take 1ml supernatant and pass it through 0.2 μm needle filter into 2 mL liquid phase vial. After sample preparation, a UHPLC-CAD analysis was performed on the Thermo Vanquish Flex UHPLC system (ThermoFisher Scientific, Waltham, MA, USA).The separation of saponins in *G. longipes* extract was achieved by using a Waters ACQUITY™ UPLC HSS T3 column (100 mm × 2.1 mm, 1.8 µm) eluted by a mobile phase consisting of 0.1% formic acid-water (A) and acetonitrile (B) with the following gradient program: 0–2 min, 10-25% B; 2–7 min, 25-32% B; 7–10 min, 32-33% B; 10-12min, 33–35% B; 12–18 min, 35-43% B; 18–22 min, 43-60% B; 22–22.5 min, 60-98% B; 22.5–26.5 min, 98% B; 26.5–27 min, 98-10% B; 27–30 min, 10% B. The flow rate was set at 0.5 mL/min, and the injection volume was 5 µL. The column temperature was maintained at 40°C, the evaporator temperature of CAD was 35°C, the filtration constant (filter) was 1 s and the data collection rate was 10 Hz.

After the samples were detected by ultrahigh performance liquid chromatography-charged aerosol detector (UHPLC-CAD), and then the peak areas of main gypenosides (gypenosides XLIX and A, malonylgypenosides XLIX and A) were recorded. In practical application, malonylgypenosides is transformed into corresponding gypenosides. Therefore, the content of gypenosides XLIX represents the sum of the peak areas of gypenoside XLIX and malonylgypenoside XLIX, the content of gypenosides A represents the and the sum of peak areas of gypenoside A and malonylgypenoside A. This study only focused on the total content of secondary metabolites. Therefore, the peak areas of gypenosides A and gypenosides XLIX were added as the relative full gypenosides content of *G. longipes*, which was used to compare the relative content of gypenosides for three provenances at two altitudes.

### 2.3 Fine root morphological characteristics

For fine roots samples, the volume of each soil sample square was divided into two uniform ones according to the depth of every 10 cm. Therefore, for two altitudes and three provenances, a total of 60 soil blocks were obtained. The soil blocks were placed into plastic bags and then washed gently by running water through sieve nets to get them clean. Live roots were distinguished from dead roots according to the difference in root shape, elasticity and color (Freschet and Roumet, 2017; Wang et al., 2019). All live fine roots were transported to the laboratory, stored at – 20°C, and then processed for morphological trait analysis.

The fine root samples were arranged on a transparent plate and scanned using an Epson Perfection V850 Pro scanner at a resolution of 400 dpi. Scanned images were analyzed for root length, root surface area, root projected area, and root volume using the WinRHIZO image analysis software (Regent Instruments Inc., Quebec, Canada). Fine root length density (FRLD, m m^− 3)^ and fine root surface area (FRSA, m^2^ m^− 3^) were calculated as root mass, root length, and root surface area per soil block volume, respectively. Specific root length (SRL, m g^-1^) was calculated as root length per unit root dry mass. Fine root averaged diameter (FRAD, mm) was the ratio of the total projected area to the total root length.

### 2.4 Soil properties

Soil samples collected from the soil blocks by 10 cm intervals in 0-20 cm soil depths at the end of October 2021 were used to determine soil properties, including pH, alkali-hydrolyzed nitrogen (AN, mg kg^−1^), available phosphorus (AP, mg kg^−1^), available potassium (AK, mg kg^−1^), soil organic matter (OM, g kg^−1^), the proportion of soil clay (Clay, %), the proportion of soil silt (Silt, %) and the proportion of soil sandy (Sandy, %). We collected soil samples through the 2 mm sieve and made them air-dried for the soil physical and chemical property parameters. A laser particle size analyzer determined the composition of soil particles. Soil pH was determined using a pH meter at a 1:5 soil/water ratio. The AN, AP, AK, and OM of soil were measured using the procedure described by He et al. (2022).

### 2.5 Statistical analysis

A Kolmogorov-Smirnov test and a Levene test were used to verify the assumptions of normality and the homogeneity of variances for all data of soil characteristics, root morphological characteristics, and plant biomass of different organ indicators. Analysis of variance (ANOVA) was performed to compare whether there were significant differences among three provenances for fine root morphological characteristics, plant biomass, and gypenosides content of different organs. Independent *t*-tests were used to compare whether there were significant differences between the two altitudes in the characteristic parameters of different organ’s fine root morphology, plant biomass, and gypenosides content. These statistical analyses were conducted with SPSS software (v. 20.0 SPSS Inc., Chicago, USA).

Redundancy analysis (RDA) was conducted to determine the relative importance of soil properties contributing to fine root morphological characteristics, fine root morphological characteristics contributing to plant biomass and gypenosides content of different organs. This analysis was used to determine which factors affected fine root characteristics, biomass, and gypenosides content, and whether the relationship between them was a positive or negative response (He et al., 2022). Relative importance metrics of soil predictors of fine root morphological characteristics and fine root morphological characteristics of biomass, and gypenosides content were calculated using the R- package relaimpo (Dang et al., 2020; Li et al., 2021). We calculated relative importance using the LMG (Lindeman, Merenda, and Gold) method that calculates the R^2^ contribution averaged over orderings among regressors. This analysis was used to evaluate how the factors affected the variables quantitatively. All the above analysis was performed using the statistical software R (R Development Core Team, 2017).

Exponential correlations were used to identify the effects of significant soil factors on fine root morphological characteristics. The exponential correlation was also used to test the relationship between fine root biomass to leaf biomass ratio and total plant biomass (TPB) for all provenances in two altitudes. These statistical analyses and all graphics were performed with the Origin 2018 software (OriginLab, USA).

## 3 Results

### 3.1 Different organ biomass and gypenosides content of *Gynostemma longipes* cultivated at two altitude habitats

**Figure 1:**
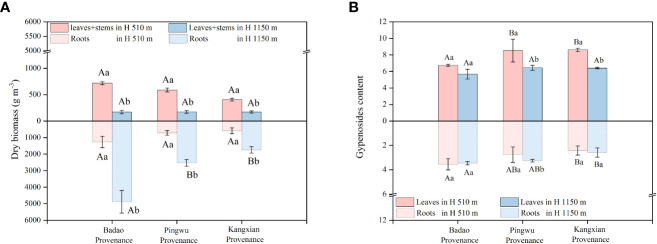
Different organ biomass **(A)** and gypenosides content **(B)** of *Gynostemma longipes* from three provenances at altitude 510 m (H_1_) and altitude 1150 m (H_2_). Different lowercase letters for the same provenances in the same organ indicate significant differences of different altitudes at α = 0.05. Different capital letters for the same organ in the same altitude indicate significant differences of different provenances at α = 0.05.

For the plants of each provenance, the plants have significantly higher absolute growth of aboveground organs growing in low-altitude habitats than in high-altitude habitats, which higher proportions was 120%, 90%, and 140% for Badao, Pingwu, and Kangxian provenance, respectively (**Figure 1A**). However, the absolute growth of underground organs in different altitude habitats had opposite results, different from the growth of aboveground organs. Specifically, the plants had significantly higher absolute growth of underground organs growing in high-altitude habitats than in low-altitude habitats, which higher proportions was 290%, 250%, and 200% for Badao, Pingwu, and Kangxian provenance, respectively. Because the absolute growth of underground organs accounted for most of the total growth of plants (59% - 97%), the total absolute growth of plants growing in high-altitude habitats was much higher than that in low-altitude habitats, which higher proportions was 210%, 160% and 90% for Badao, Pingwu and Kangxian provenance, respectively.

In addition, there was no significant difference between provenances in aboveground and absolute underground growth for plants growing in low-altitude habitats. The absolute growth of aboveground organs had no significant difference among provenances for plants growing in high-altitude habitats. However, the absolute growth of underground organs was markedly different among provenances. That is, the Badao provenance was significantly higher than the other two provenances, but there was no difference between the other two provenances.

The content of gypenosides in the aboveground and underground organs of the Badao provenance did not change with the change of altitude habitats (**Figure 1B**). However, Pingwu provenance and Kangxian provenance were different results. Specifically, the gypenosides content of the leaves of Pingwu provenance growing in the low-altitude habitat was 32% higher than that in the high-altitude habitat. Still, the gypenosides content in the roots was 18% higher in the high-altitude habitat than that in the low-altitude habitat. For Kangxian provenance, the gypenosides content of the plants growing in the low-altitude habitat was significantly 34% higher than that in the high-altitude habitat. Still, the gypenosides content of roots had no significant difference between the two altitude habitats.

In addition, for plants growing in low-altitude habitats, the gypenosides content of the leaves were 6.74 ± 0.13, 8.53 ± 1.38 and 8.61 ± 0.20 for Badao, Pingwu, and Kangxian, respectively (**Figure 1B**). However, for the gypenosides content of the roots, plants growing at low-altitude and high-altitude habitats had similar variation trends among the three provenances: Badao (H_1_ 3.55 ± 0.47; H_2_ 3.46 ± 0.13) > Pingwu (H_1_ 2.76 ± 0.64; H_2_ 3.25 ± 0.11) > Kangxian (H_1_ 2.42 ± 0.38; H_2_ 2.59 ± 0.38).

### 3.2 Fine root distribution and root morphological characteristics of *Gynostemma longipes*

For *G. longipes* from three provenances growing at two altitudes, the roots were distributed in the topsoil (0-20 cm), a shallow root vine. Specifically, 80% of fine roots were distributed at 0-10 cm and about 20% at 10-20 cm (Data not shown). The distribution proportion of fine roots in different soil layers was quite similar for different altitude habitats. Therefore, we combined the roots of 10 cm and 20 cm soil layers for the following analysis.

**Figure 2:**
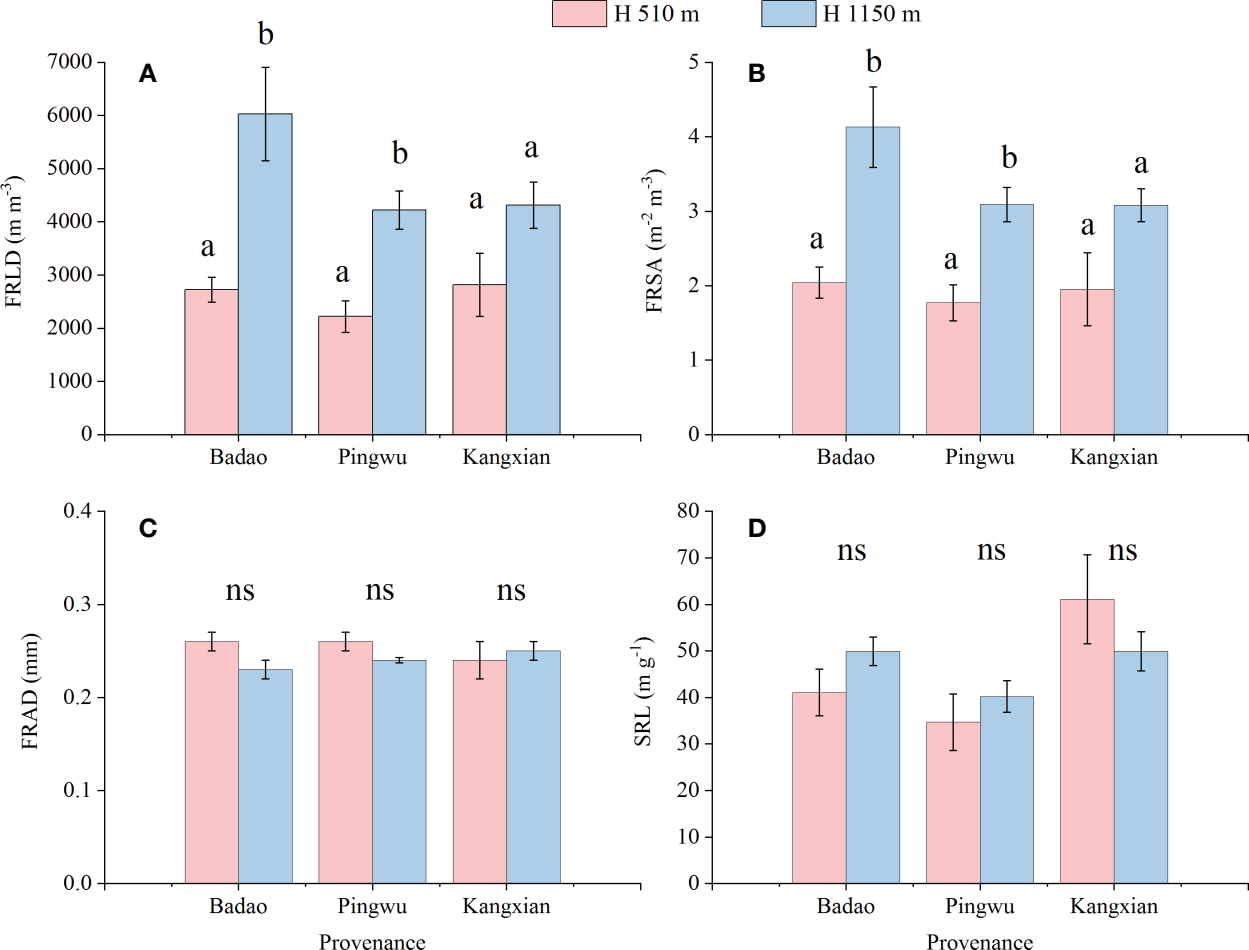
Fine root characteristics: fine root length density (FRLD) **(A)**, fine root surface density (FRSA) **(B)**, fine root average diameter (FRAD) **(C)** and specific root length (SRL) **(D)** of *Gynostemma longipes* from three provenances at altitude 510 m (H_1_) and altitude 1150 m (H_2_). Different lowercase letters for the same provenances indicate significant differences of different altitudes at α = 0.05. “ns” for the same provenances indicates that no significant difference of different altitudes at α = 0.05.

Fine root morphological characteristics (i.e., FRLD, FRSA, FRAD, SRL) were significantly changed by altitude habitats for Badao and Pingwu provenances plants (**Figures 2A, B**). Specifically, the FRLD of plants growing in high-altitude habitats were significantly higher than that in low-altitude habitats by 121% and 61%, for Badao provenance and Pingwu provenance, respectively. The FRSA of plants growing in high-altitude habitats were significantly higher than that in low-altitude habitats by 103% and 90% for Badao provenance and Pingwu provenance, respectively. But for Kangxian provenance, the altitude habitat did not significantly change the FRLD and FRSA (**Figures 2A, B**). altitude habitats did not considerably change FRAD and SRL of all three provenances plants (**Figures 2C, D**). Besides, for the plants growing in the same altitude habitat, there was no significant difference among the three provenances in fine root characteristics (*p* > 0.05) (result not shown).

### 3.3 Different organ biomass and gypenosides content of *Gynostemma longipes* response to fine root morphological characteristics

**Figure 3:**
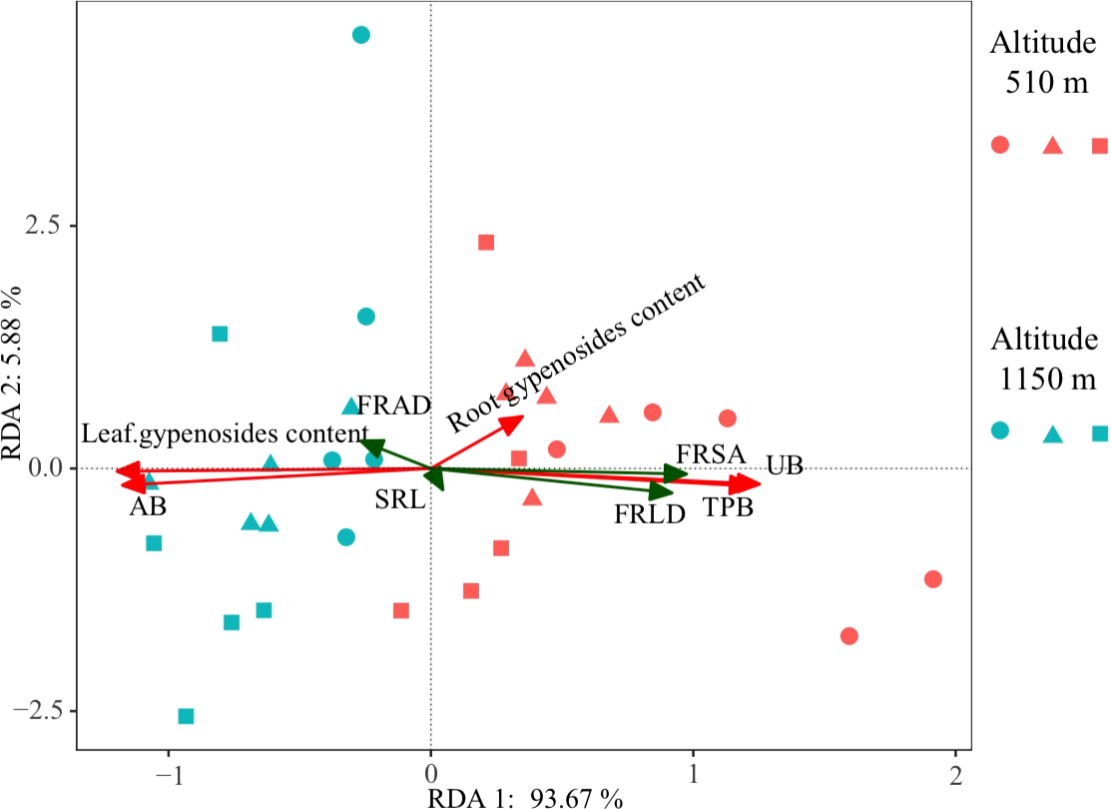
Redundancy analysis (RDA) of biomass and gypenosides content and fine root characteristics for *Gynostemma longipes* from three provenances at altitude 510 m (H_1_) and altitude 1150 m (H_2_) (n = 30). Red arrows represent growth and quality parameters of *G. longipes* (AB, UB, TPB, leaf gypenosides content and root gypenosides content), green arrows represent fine root traits (FRLD, FRSA, FRAD and SRL). Abbreviations of biomass and fine root characteristics are as follows: AB, aboveground biomass (g m^-2^); UB, underground biomass (g m^-2^); TPB, total plant biomass (g m^-2^); FRLD, fine root length density (m m^−3^); FRSA, fine root surface area (m^2^ m^−3^); FRAD, fine root averaged diameter (mm); SRL, specific root length (m g^−1^). Circle, triangle and square represent the data from Badao provenance, Pingwu provenance and Kangxian provenance, respectively.

RDA results showed that the main fine root characteristics affecting UB and TPB were similar. FRLD and FRSA were the main positive factors of UB and TPB (**Figure 3**). However, for AB and leaf gypenosides, FRLD and FRSA were the main negative factors. The fine root characteristics explained 99.55% of the total variation, with axes 1 and 2 defining 93.67% and 5.88% of the total variation, respectively (**Figure 3**). The RDA results were also confirmed by relative importance analysis, which was used to quantitatively describe the impact of each fine root characteristics on growth and gypenosides content of *G. longipes* (**Table 1**). Our results showed that growth of different organs and leaf gypenosides content could be significantly described by fine root characteristics, and R^2^ were 0.51, 0.43, 0.48 and 0.30 for UB, AB, TPB, and leaf gypenosides content, respectively. However, fine root characteristic parameters could not significantly describe the root gypenosides content (*P* > 0.05). The relative importance of the main factors affecting growth (UB, AB, and TPB) was consistent, and the order of priority was FRSA (0.49, 0.52, 0.49) and FRLD (0.48, 0.44, 0.48). FRLD and FRSA were also the main factors affecting the leaf gypenosides; their relative importance was equal, both of which were 0.48 (**Table 1**).

**Table 1:** Relative importance metrics of growth and gypenosides content of *Gynostemma longipes*: AB, aboveground biomass (g m^-2^); UB, underground biomass (g m^-2^); TPB, total plant biomass (g m^-2^); leaf gypenosides content; root gypenosides content.

Response	P	R^2^	FRLD	FRSA	FRAD	SRL
UB	< 0.001	0.51	0.48***	0.49***	0.01	0.02
AB	< 0.001	0.43	0.44***	0.52***	0.01	0.04
TPB	< 0.0001	0.48	0.48***	0.49***	0.01	0.02
Leaf gypenosides	< 0.05	0.30	0.48***	0.48***	0.01	0.03
Root gypenosides	> 0.05	–	–	–	–	–

The *** represented significant correlations between the fine root morphology index (FRLD/FRSA) and the response variables at α = 0.001.

**Figure 4:**
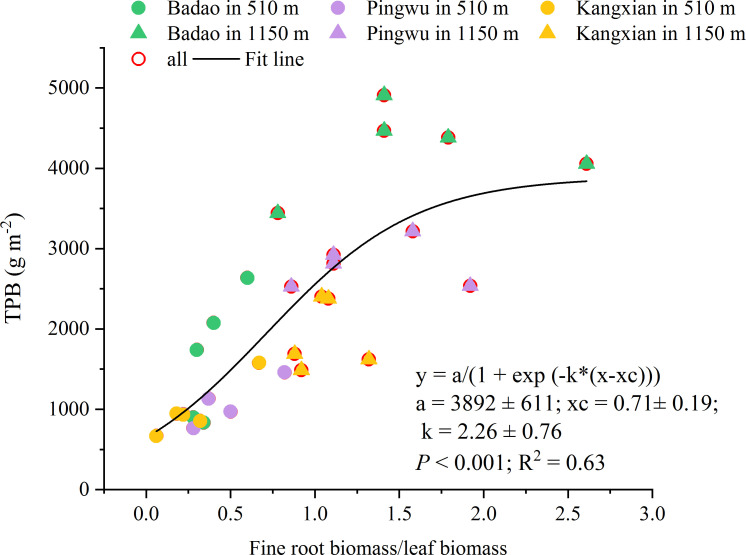
The relationship between fine root biomass to leaf biomass ratio and total plant biomass (TPB) for all provenances in two altitudes.

The total plant biomass significantly increased with the increment of the fine root biomass to leaf biomass among all three provenances (**Figure 4**). It indicated that the harvest yield of the total plant could be effectively increased by promoting the growth of fine roots per unit leaf weight.

### 3.4 Fine root morphological characteristics response to soil physical and chemical properties

**Figure 5:**
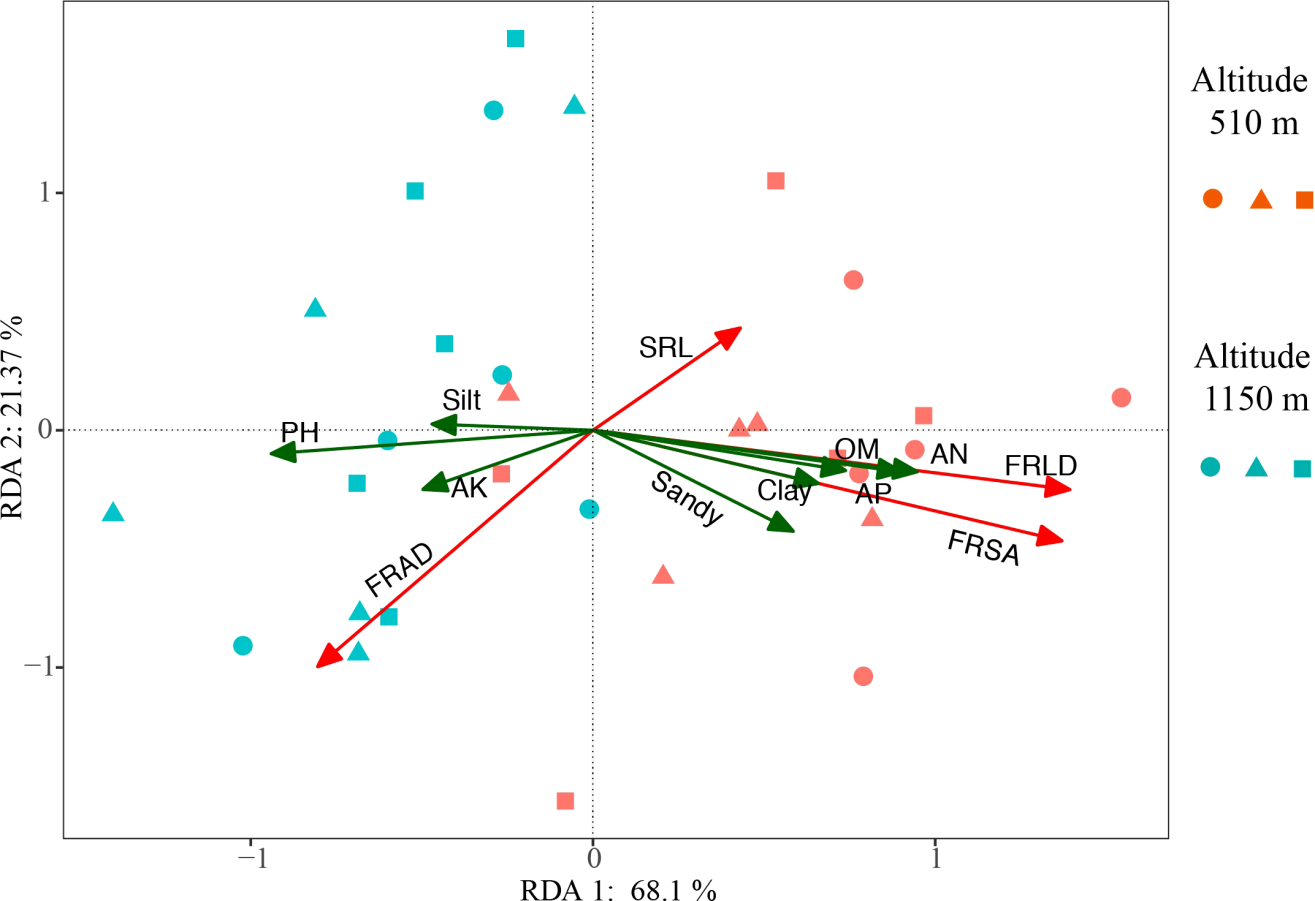
Redundancy analysis (RDA) of fine root characteristics and soil properties for *Gynostemma longipes* from three provenances at altitude 510 m (H_1_) and altitude 1150 m (H_2_) (n = 30). Red arrows represent four fine root traits (FRLD, FRSA, FRAD and SRL), green arrows represent soil properties (AN, AP, OM, pH, AK, Clay, Silt and Sandy). Abbreviations of fine root traits and soil properties are as follows: FRLD, fine root length density (m m^−3^); FRSA, fine root surface area (m^2^ m^−3^); FRAD, fine root averaged diameter (mm); SRL, specific root length (m g^−1^); AN, Alkali hydrolyzed nitrogen (mg kg^−1^); AP, available phosphorus (mg kg^−1^); AK, available potassium (mg kg^−1^); OM, soil organic matter (g kg^−1^); Clay, proportion of soil clay (%); Silt, proportion of soil silt (%); Sandy, proportion of soil sandy (%). Circle, triangle and square represent the data from Badao provenance, Pingwu provenance and Kangxian provenance, respectively.

The soil physical and chemical properties in the two altitude habitats are shown in **S-Table 1**. The pH in H_1_ was significantly higher than in H_2_, but the soil nutrient factors (AN, OM, AP) in H_2_ were considerably higher than in H_1_. In addition, Soil in H_2_ also had a higher content of sand and clay than in H_1_. These soil factors were explained the variation of fine root characteristics at two altitudes. RDA results showed that the main soil factors affecting FRLD and FRSA were similar. The positive correlation factors were OM, AN, and AP, and the negative correlation factor was pH (**Figure 5**). They accounted for significant proportions of the variation in the fine root trait syndromes under different altitude habitats. The soil properties explained 89.47% of the total variation, with axes 1 and 2 defining 68.1% and 21.37% of the total variation, respectively (**Figure 5**). The RDA results were also confirmed by relative importance analysis, which was used to quantitatively describe the impact of each soil factor on fine root characteristics (**Table 2**). Our results showed that the relative importance of the main factors affecting FRLD and FRSA was consistent. The order of importance was AN (0.28, 0.27), OM (0.18, 0.17), AP (0.17, 0.17), and pH (0.17, 0.15). The most important factor affecting FRAD was pH, followed by Sandy, AK, AP and Clay.

**Table 2:** Relative importance metrics of fine root characteristics: fine root length density (FRLD), fine root surface density (FRSA), fine root average diameter (FRAD) and specific root length (SRL).

Response	pH	AN	OM	AP	AK	Clay	Silt	Sandy	P	R^2^
FRLD	0.1651***	0.2811**	0.1837*	0.1681**	0.0215	0.0736	0.0216	0.0849	< 0.0001	0.5786
FRSA	0.1470***	0.2705**	0.1693*	0.1691**	0.0417	0.0917	0.0227	0.0876	< 0.0001	0.5738
FRAD	0.2992**	0.0604	0.0346	0.1027**	0.1666**	0.1012**	0.0578	0.1771**	< 0.05	0.3921
SRL	–	–	–	–	–	–	–	–	> 0.05	–

The "***", "**", and "*" represented significant correlations between the soil indicators and the response variables at α = 0.001, 0.01, and 0.05, respectively.

**Figure 6:**
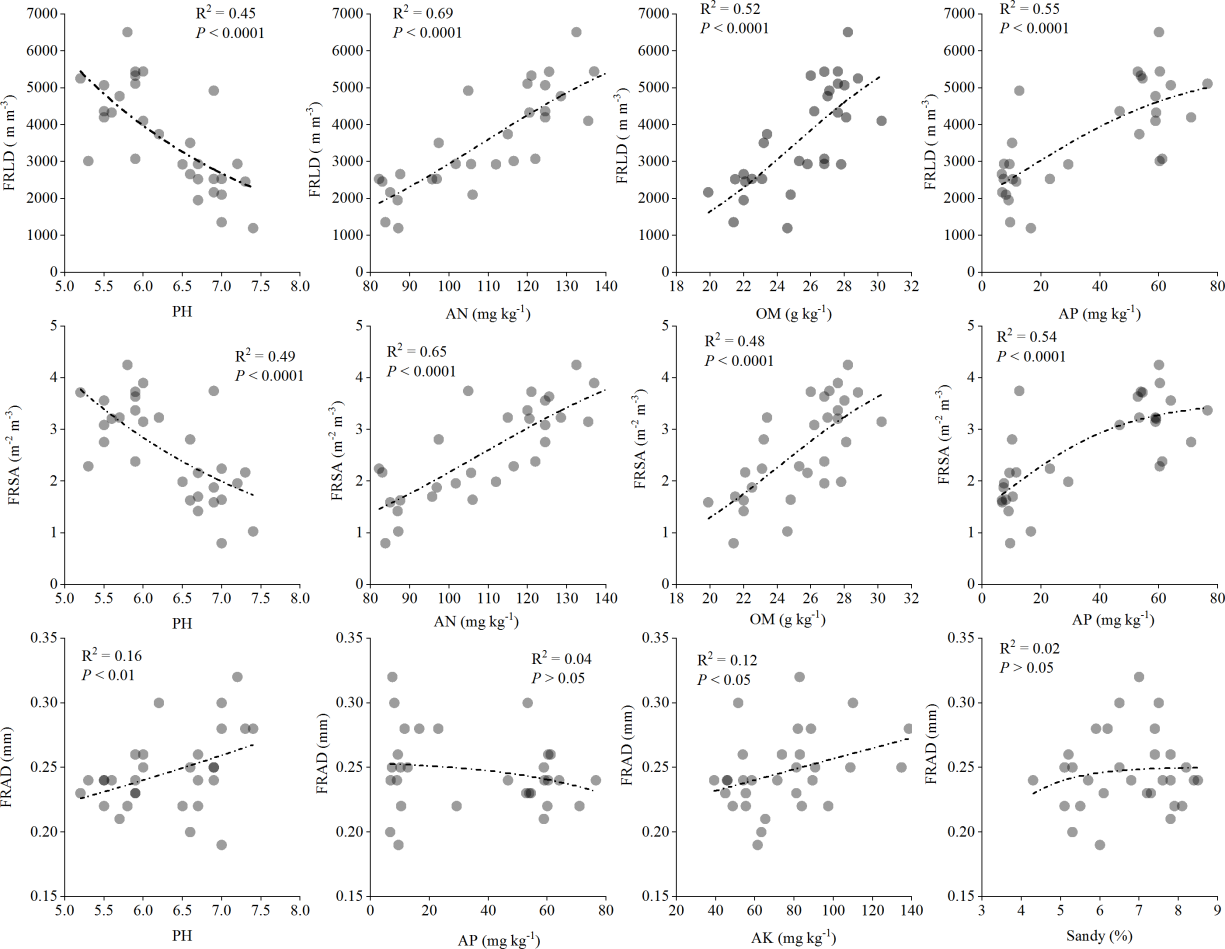
Quantitative relationship between fine root characteristics and main soil factors for *Gynostemma longipes* from three provenances at altitude 510 m (H_1_) and altitude 1150 m (H_2_) (n = 30). Abbreviations of fine root traits and main soil factors are as follows: FRLD, fine root length density (m m^−3^); FRSA, fine root surface area (m^2^ m^−3^); FRAD, fine root averaged diameter (mm); AN, Alkali hydrolyzed nitrogen (mg kg^−1^); AP, available phosphorus (mg kg^−1^); AK, available potassium (mg kg^−1^); Sandy, proportion of soil sandy (%). The black dotted line represents the best fit line between fine root characteristics and soil factors.

We also studied the quantitative relationship between the main factors affecting the characteristics of fine roots and the morphological characteristics of fine roots (**Figure 6**). The results showed that pH had a strong negative correlation with FRLD (*p* < 0.0001, R^2^ = 0.45), while AN (R^2^ = 0.69), OM (R^2^ = 0.52) and AP (R^2^ = 0.55) had a strong positive correlation with FRLD, respectively. Similarly, FRSA was also strongly negatively correlated with pH (R^2^ = 0.49) and strongly positively correlated with AN (R^2 =^ 0.65), OM (R^2^ = 0.48), and AP (R^2^ = 0.54). However, there was a significant positive correlation between FRAD and pH (*p* < 0.01, R^2^ = 0.16) and between FRAD and AK (*p* < 0.05, R^2^ = 0.12).

## 4 Discussion

*Gynostemma longipes* plant has become a famous medicinal plant in Eastern countries due to its active ingredients similar to ginsenosides. However, the lack of precise field cultivation and mechanism research on medicinal plants, including *G. longipes*, limits their yield and quality stability. As a critical functional organ for plants to absorb water and nutrients from the soil, fine roots play an essential role in forming plant yield and quality. Therefore, in this study, we explored how the growth and gypenosides content of medicinal *G. longipes* varied with altitude habitats, as well as the main plant factors (fine root morphological characteristics) and environmental factors (soil factors) that caused this change. To achieve this overall goal, we sequentially studied (1) the growth and gypenosides content changes of *G. longipes* in two altitude habitats; (2) The relationship between growth, gypenosides, and fine root morphological characteristics of *G. longipes*, and (3) The main soil factors affecting the fine root morphology of *G. longipes*. This is the first time that systematic study has been established on medicinal plants, ranging from plant growth and quality to plant morphological characteristics (fine root characteristics) to environmental impacts (soil factors), which has guiding significance for achieving precise field cultivation research medicinal plants. Therefore, we will also discuss these three small research objectives below.

### 4.1 Plant growth and secondary metabolism accumulation at different altitude habitats

Studying the characteristics of medicinal plant growth and secondary metabolites (SMs) in different altitude habitats is very important for carrying out targeted ecological planting technology to improve the yield and quality of medicinal plants. Our results showed that at the end of the growing season, for all provenances, with rising altitude, the aboveground biomass decreased. In contrast, the underground biomass increased significantly (**Figure 1A**). This may be due to the lower temperature and shorter growing season length in high-altitude habitat than the lower site, which promoted the high growth of roots (Feßel et al., 2016). Our findings were also consistent with *Gentiana rhodantha* on the Yunnan-Guizhou Plateau (Zhang et al., 2020), grasslands of Tibet (Bhandari and Zhang, 2019), and herbage on Mt Varnoudas, NW Greece (Mountousis et al., 2011). Interestingly, our results also showed that the growth of *G. longipes* from the three provenances maintained similar response laws to the altitude habitat variation. This showed that the growth characteristics of *G. longipes* of different provenances were mainly determined by their natural living environment rather than their genetic specificity. However, the extent of biomass change with altitude increase varied with provenance. Whether for root or leaf biomass, the yield of Badao provenance under suitable habitats was better than that of the other two provenances. This may be since the Badao provenance is the native provenance of the study area, which can maximize the growth potential in the suitable microhabitat area after long-term climate adaptation.

The accumulation of secondary metabolites is a crucial evaluation factor for the ecological planting technology of medicinal plants, and it is essential to select the most suitable habitat for medicinal plants. Our study showed that the response of gypenosides content to altitude habitats differed according to provenances and plant organs (**Figure 1B**). Specifically, in our research results, leaf gypenosides content was higher at low-altitude habitats. In comparison, root gypenosides content was higher at high-altitude habitats. This showed that the environmental stress factors of gypenosides content in different organs of *G. longipes* were various. This phenomenon was also reported in *Panax notoginseng* (Zhan et al., 2022), *Paris polyphylla* (Qiang et al., 2020), and *Sapindus* (Liu et al., 2022). The most important reason for the complex response of SMSs to altitude habitat factors may result from a long-term adaptation of different provenances to native habitats (Berini et al., 2018; Yang et al., 2018; Li et al., 2020).

From these findings, we could understand that altitude habitat significantly impacted the yield of *G. longipes* from different provenances, consistent with our first hypothesis. However, the response of gypenosides content to different altitude habitats varied with provenance and plant organs. Therefore, in future studies, we should further study the biological and abiotic factors that affect the gypenosides content to clarify the mechanism of its gypenosides content.

### 4.2 Relationship between functional characteristics of fine roots and plant growth and quality

Fine roots are the main foraging organs of plants, so plants absorb water and nutrients from the soil through fine roots to meet the accumulation of primary and secondary metabolites (Canarini et al., 2019; Nikolova et al., 2020; Wang et al., 2020). Therefore, many studies have reported that fine root characteristics were directly related to plant yield and quality (Rondina et al., 2020; Song et al., 2020; Sun et al., 2020). Our work showed that, through RDA and relative importance analysis, FRLD and FRSA were the most critical fine root characteristic parameters that affect the primary and secondary metabolites of *G. longipes*, which explains about 90% of UB, AB, TPB, and gypenosides content (**Figure 3** and **Table 1**). As far as we know, no one has reported such a quantitative relationship between fine root characteristics and growth & gypenosides in *G. longipes*. This is very important for understanding the ecophysiological basis of forming the yield and quality of *G. longipes* in the future.

Thus, our second hypothesis, i.e., there is a quantitative relationship between the growth and quality formation of medicinal *G. longipes* and some parameters of fine root characteristics in different altitude habitats, is partially accepted. In our study, the significant relationship between FRLD and FRSA and growth& gypenosides is because we have a wide range of fine root morphological characteristics and biomass variation. This is because the fine root morphological characteristics of FRLD and FRSA show significant differences in different altitude habitats (**Figures 1**, **2**). However, the fine root morphological characteristics FRAD and SRL do not change with the altitude habitat, which indicates that the fine root characteristics of *G. longipes* result from the interaction of genetics and environment. Zou et al. (2019) also showed that FRLD and FRSA were more susceptible to environmental factors, while FRAD and SRL were more genetically determined. However, these studies are only based on the data at the end of a growth season. With the different growth stages of plants, the relationship between the morphological characteristics of fine roots and growth and quality may also change. Therefore, to better understand the change rule of fine root characteristics and its quantitative relationship with the yield and quality of *G. longipes*, it is necessary to explore the relationship between the fine root characteristics, growth, and quality of *G. longipes* at different growth stages and different ages.

### 4.3 Response of plant fine root characteristics to soil parameters

Previous studies had shown that the fine root morphological characteristics of most plants were affected by both genes and environmental factors, while soil factors (texture, water nutrient status, etc.) had much more influence on it than the control of genes (Weemstra et al., 2017; Brunner et al., 2019; He et al., 2022; Lu et al., 2022). Fine roots can enhance the absorption of environmental resources by increasing biomass or improve the absorption and utilization efficiency of environmental resources by changing the morphological characteristics of fine roots (Li et al., 2021). Many studies have shown that soil’s physical and chemical properties were essential factors affecting the morphology and growth of fine roots of plants (He et al., 2022; Zou et al., 2022). Likewise, in our study on the growth of *G. longipes* from three provenances in two altitude habitats, soil alkali hydrolyzable nitrogen (AN), available phosphorus (AP), organic matter (OM), and pH significantly affected the fine root characteristics (**Figures 5**, **6**, **Table 2**). Thus, our third hypothesis is accepted, i.e., a significant correlation between soil-related parameters and fine root characteristics of *G. longipes*.

Consistent with the research on the response of fine root characteristics to soil factors in crops and woody trees (Mulia and Dupraz, 2006; Steinemann et al., 2015; Hirte et al., 2018), our research results showed that the fine root morphological characteristics, FRLD and FRSA showed significant positive linear correlations with AN and OM (**Figure 6**). However, it is inconsistent that FRLD showed an S-shaped curve of first stability and then increased with OM and AN increase (Di et al., 2013). In addition, the response of fine root FRLD and FRSA of *G. longipes* to AP in soil presented an S-shaped curve consistent with the poplar fine roots of Di et al., 2013. The above results indicate that in our research habitats, the soil AP up to 80 mg/kg could meet the growth of fine roots of *G. longipes*, while the soil AN and OM were far from meeting the growth of fine roots of *G. longipes*. Therefore, in future research, it is essential to set up a particular nutrient addition experiment to explore the best soil nutrient level required for fine root growth of *G. longipes*.

Our research results show that soil nutrients, rather than soil physical structure, are the main factors affecting the growth of fine roots of *G. longipes*. However, the above results indicated that the response of *G. longipes* fine roots to soil nutrient factors varied with plant varieties and growth stages. Future research should study the main soil factors of *G. longipes* fine root growth in a broader spatial scale and more growth stages.

## 5 Conclusions

This study assessed how the growth and gypenosides of *Gynostemma longipes* changed with the altitude habitats. We found that the *G. longipes* had significantly higher absolute growth of underground organs in high-altitude habitats than in low-altitude habitats, with more than 200% higher proportion for all three provenances. The growth of *G. longipes* was significantly explained by fine root morphological indicators (fine root length density and fine root surface area) (*P* < 0.001), and the fine root characteristics of *G. longipes* were significantly controlled by soil factors, especially nutrient factors (alkali-hydrolyzed nitrogen, soil organic matter, available phosphorus). Our results significantly improved our understanding of the ecophysiology basis for forming the yield and quality of *G. longipes* under changing habitat conditions. This finding helps clarify the optimal habitat threshold and provides a strong guarantee for precise cultivation of lipid-lowering drugs using the root of *G. longipes* as an essential raw material to solve the current situation of shortage of wild resources of *G. longipes*. In future research, we should clarify the main plant (e.g., fine root characteristics) and environmental (e.g., soil factors) controlling the yield and gypenosides of *G. longipes* at a longer time scale.

## Author contributions

DL, GL, JG, DW, FC and JL performed the field and laboratory experiments. DL and BX analyzed the data. DL and GL wrote the manuscript. DL, BM and BG revised and finalized the manuscript. All authors contributed to the article and approved the submitted version.
