# Supplementary material for: Response mechanism of growth and gypenosides content for Gynostemma longipes cultivated at two altitude habitats to fine root morphological characteristics
Source: Front Plant Sci. 2023 May 30;14:1143745. doi: 10.3389/fpls.2023.1143745 (PMC10265677; doi:10.3389/fpls.2023.1143745)
Supplement: Supplementary file 1 [file Table_1.docx]

S-Table 1 Soil physical and chemical properties at two altitude habitats: AN, Alkali hydrolyzed nitrogen (mg kg^−1^); AP, available phosphorus (mg kg^−1^); AK, available potassium (mg kg^−1^); OM, soil organic matter (g kg^−1^); Clay, proportion of soil clay (%); Silt, proportion of soil silt (%); Sandy, proportion of soil sandy (%).

| Experimental site | Provenance | pH | AN  (mg kg^-1^) | OM  (g kg^-1^) | AP  (mg kg^-1^) | AK  (mg kg^-1^) | Clay  % | Silt  % | Sandy  % |
| --- | --- | --- | --- | --- | --- | --- | --- | --- | --- |
| Low altitude  (H_1_ 510 m) | Badao | 6.8 (±0.1) | 100.3 (±2.3) | 23.6(±0.8) | 9.0(±0.6) | 100.5(±9.9) | 4.1(±0.1) | 46.3(±0.1) | 5.5(±0.3) |
|  | Pingwu | 7.0 (±0.2) | 90.2(±5.5) | 23.9(±1.1) | 17.9(±3.7) | 93.0(±13.1) | 4.2(±0.1) | 46.3(±0.8) | 6.1(±0.3) |
|  | Kangxian | 6.9 (±0.1) | 92.6(±4.5) | 23.4(±1.5) | 8.6(±1.1) | 74.1(±9.9) | 4.5(±0.3) | 46.3(±0.6) | 5.8(±0.5) |
| High altitude  (H_2_ 1150 m) | Badao | 5.7 (±0.1) | 128.7(±2.8) | 27.3(±0.4) | 59.5(±1.6) | 57.8(±5.8) | 5.1(±0.0) | 45.8(±0.4) | 7.6(±0.2) |
|  | Pingwu | 5.5 (±0.1) | 128.7(±5.5) | 27.6(±0.9) | 55.9(±2.5) | 73.8(±7.5) | 5.0(±0.1) | 45.1(±0.2) | 7.4(±0.4) |
|  | Kangxian | 5.9 (±0.1) | 121.4(±1.9) | 26.5(±0.8) | 63.0(±4.7) | 49.0(±1.7) | 5.3(±0.2) | 44.8(±0.2) | 7.8(±0.2) |
